# Favourable outcome of pathologic downstaging by locoregional treatment for hepatocellular carcinoma in liver transplantation

**DOI:** 10.1038/s41598-019-46871-9

**Published:** 2019-07-17

**Authors:** Deok Gie Kim, Jae Geun Lee, Dong Jin Joo, Soon Il Kim, Myoung Soo Kim

**Affiliations:** 1 Department of Surgery, Yonsei University Wonju College of Medicine, Wonju, Republic of Korea; 2 Department of Surgery, Yonsei University College of Medicine, Seoul, Republic of Korea; 3 The Research Institute for Transplantation, Yonsei University College of Medicine, Seoul, Republic of Korea

**Keywords:** Liver, Hepatocellular carcinoma

## Abstract

No distinct guidelines are available regarding the effect of pretransplant locoregional treatment (LRT) in hepatocellular carcinoma (HCC) staging system. The aim of this study was to investigate the prognosis of pathologic downstaging (PDS) by the exclusion of total necrosis after liver transplantation. We conducted a study of 326 HCC patients who underwent liver transplantation between September 2005 and December 2016. Two hundred twenty-two patients received pretransplant LRT and 102 patients did not. Among the former group, 74 (33.0%) achieved PDS while 150 (67.0%) showed unchanged T stage after the exclusion of total necrosis. Five-year HCC recurrent free survival (RFS) of PDS group (85.1%) was similar to that of the no LRT group (88.8%) but higher than that of the non-PDS group (68.9%; *P* < 0.001). Based on T stage adjusted with total necrosis and PDS status, RFS was similar in the PDS T1 (82.4%) and non-PDS T1 (86.5%) groups. Non-PDS T2 cancers had worse outcome regardless of the Milan (*P* = 0.982) or University of California San Francisco criteria (*P* = 0.466). On preoperative examination, parameters like less than 1 viable tumor, less than 1 cm of tumor size, and less than 20 ng/mL of serum alpha fetoprotein were associated with PDS. This study showed that PDS by LRT was associated with favorable outcome in HCC patients after liver transplantation.

## Introduction

Liver transplantation (LT) has been a primary treatment option for unresectable hepatocellular carcinoma (HCC) since the Milan criteria was introduced^1^. There have been ongoing attempts to expand the indication of LT^2,3^. Moreover, locoregional treatment (LRT) was applied to patients with large tumour burden for achieving equivalent outcome as those initially within the criteria for LT^4,5^. Several studies estimated the effectiveness of LRT based on the radiologic criteria^6,7^. However, discrepancy up to 25% was reported between radiologic assessment before LT and explant liver pathology^8^. Precise prediction and early detection of tumour recurrence after LT is desirable to achieve the best treatment outcome^9^. Heretofore, the method of consensus for determining the prognosis of HCC after LT was the American Joint Committee on Cancer staging system^6^, although other recurrence prediction models based on explant pathology have been developed^10^. However, none of the models reflected the accurate result of LRT, and there was no distinct guideline about considering each tumour necrosis on the explanted liver. Hence, the aim of this study was to investigate the consequence of pathologic downstaging (PDS; reduced T stage by the exclusion of totally necrotic mass) in terms of post-transplant outcomes for HCC.

## Results

Of 326 recipients with HCC, 220 (67.5%) and 106 (32.5%) received living and deceased donor LTs, respectively. The underlying liver diseases were hepatitis B (n = 262, 80.4%), hepatitis C (n = 26, 8.0%), alcoholic liver disease (n = 22, 6.7%), and others (n = 16, 4.9%). Among the study population, 224 patients (68.7%) received pretransplant LRT, whereas 102 (31.3%) did not. During a median follow-up of 54.5 (interquartile range [IQR] 28.0–84.5) months, 41 (12.6%) patients experienced HCC recurrence. At the last follow-up date, April 30^th^, 2018, 14 (4.3%) were alive with HCC recurrence, 27 (8.2%) died following HCC recurrence, and 29 (8.9%) died without HCC recurrence.

### Changes in T stage by the exclusion of total necrosis

**Figure 1:**
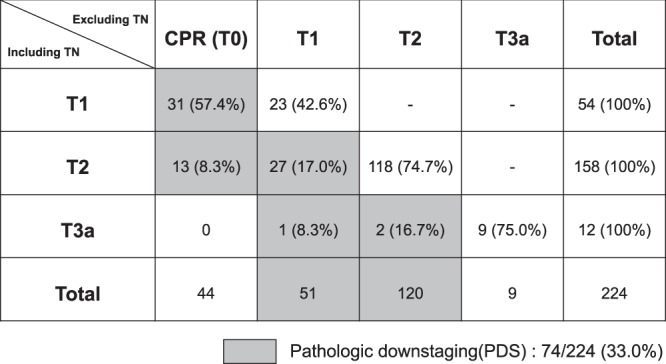
T stage changes before and after exclusion of totally necrotic mass in patients receiving pretransplant locoregional treatment; shadow denotes pathologic downstaging CPR, complete pathologic response; TN total necrosis.

Figure 1 shows changes in T stage including and excluding total necrosis (TN; tumour mass without viable cancer cell) based on the explanted liver pathology. Of 224 patients who received LRT, 44 patients achieved complete pathologic response (CPR; all tumour masses were TN) in whom the original stage was T1 (n = 31) or T2 (n = 13). The number of PDS T1 was 28, with original stage T2 (n = 27) and T3a (n = 1). Only 2 of the PDS T2 showed downstaging from the original T3a. Overall, 74 (33.0%) patients showed PDS, while 150 (67.0%) had unchanged T stage after the exclusion of TN (non-PDS).

### Clinical characteristics

Comparison of the clinical characteristics is shown in Table 1. Age, sex, body mass index, LT from living donor, and ABO incompatibility were similar. History of liver resection for HCC was more frequent in the PDS and non-PDS groups than in the no LRT group (*P* = 0.024). AFP at the time of LT was significantly lower in the PDS group than that in the non-PDS, and further lower than that in the no LRT group (*P* = 0.005). On radiologic evaluation at transplantation, the non-PDS group had more frequent multiple viable tumours and was more frequently beyond the Milan criteria than the other two groups (*P* = 0.001).

**Table 1:** Clinical characteristics.

Variables	No LRT (n = 102)	PDS (n = 74)	Non-PDS (n = 150)	*P*
Age, years	54 (50–61)	55 (49–61)	55 (51–59)	0.953
Sex, male	83 (81.4%)	55 (74.3%)	129 (86.0%)	0.101
BMI	23.5 (21.9–25.9)	24.1 (22.3–25.4)	24.2 (22.5–26.2)	0.698
History of liver resection for HCC	9 (8.8%)	18 (24.3%)	27 (18.0%)	0.020
Donor type, living	71 (69.6%)	45 (60.8%)	104 (69.3%)	0.378
ABO incompatible	8 (7.8%)	3 (4.1%)	18 (12.0%)	0.131
MELD score at transplantation	12 (8–17)	9 (7–17)	10 (7–12)	0.016
AFP, at transplantation	7 (4–26)	6 (3–14)	9 (4–39)	0.005
**Radiologic finding at LT**				
Beyond the Milan criteria	12 (11.8%)	4 (5.4%)	35 (23.3%)	0.001
Number of viable tumors				<0.001
0	27 (26.5%)	29 (39.2%)	19 (12.7%)	
1	52 (51.0%)	33 (44.6%)	66 (44.0%)	
2 or 3	20 (19.6%)	11 (14.6%)	48 (32.0%)	
≥4	3 (2.9%)	1 (1.4%)	17 (11.3%)	
Maximum tumor diameter, cm	2.0 (0–2.7)	1.0 (0–1.8)	1.8 (1.0–2.8)	<0.001
Total number of pretransplant LRT	—	2 (1–4)	2 (1–4)	0.590
Modality of LRT				0.277
TACE, only	—	44 (59.5%)	108 (72.0%)	
RFA, only	—	11 (14.9%)	13 (8.7%)	
TACE plus RFA	—	17 (23.0%)	26 (17.3%)	
Miscellaneous	—	2 (2.7%)	3 (2.00%)	

AFP alpha fetoprotein; BMI, body mass index; HCC hepatocellular carcinoma; MELD, model for end-stage liver disease; LRT, locoregional treatment; RFA, radiofrequency ablation; PDS, pathologic downstaging; TACE, trans arterial chemoembolization.

In both PDS and non-PDS groups, transarterial chemoembolization (TACE) only was the most frequent modality followed by TACE plus radiofrequency ablation (RFA). There was no difference in the type (*P* = 0.277) and the number (*P* = 0.590) of LRT between the two groups.

### Explanted liver pathology

As shown in Table 2, the PDS group showed single TN in 66.2% and multiple TN in 33.8%. In contrast, the non-PDS group showed no TN in 58.7%. The rates of single TN and multiple TN were 44.7% and 30.0%, respectively. Among the PDS group, 59.5% had no viable cancer, whereas 39.2% had solitary viable cancer. Only one case showed more than 4 viable tumours, and it was downstaged from T3a to T2. In contrast, the non-PDS group had multiple HCC in 74.7% and more than 4 viable tumours in 30.0%. The maximum tumour diameter was lower in the PDS group than that in the non-PDS and even lower than that in the no LRT group (*P* < 0.001). Microvascular invasion (MVI) and poor differentiation were lower in the PDS group than those in the other two groups (*P* < 0.001 for both variables).

**Table 2:** Explanted liver pathology.

Variables	No LRT (n = 102)	PDS (n = 74)	Non-PDS (n = 150)	*P*
Number of TN				<0.001
0	—	0	88 (58.7%)	
1	—	49 (66.2%)	30 (20.0%)	
≥2	—	25 (33.8%)	32 (21.3%)	
Number of viable tumors				<0.001
0	0	44 (59.5%)	0	
1	55 (53.9%)	29 (39.2%)	38 (25.3%)	
2 or 3	36 (35.3%)	0	67 (44.7%)	
≥4	11 (10.8%)	1 (1.4%)	45 (30.0%)	
Maximum tumor diameter, cm	2.0 (1.3–3.0)	1.0 (0–1.1)	2.2 (1.5–3.2)	<0.001
Microvascular invasion	24 (23.5%)	2 (2.7%)	51 (34.0%)	<0.001
Differentiation				<0.001
No viable tumor	0	44 (59.5%)	0	
Well	28 (27.5%)	8 (10.8%)	16 (10.7%)	
Moderate	46 (45.1%)	18 (24.3%)	77 (51.3%)	
Poor	28 (27.5%)	4 (5.4%)	57 (38.0%)	

PDS, pathologic downstaging; TN, total necrosis.

### Pretransplant predictive factors for pathologic downstaging

To define the factors associated with PDS, we performed regression analysis with patient, serologic, and radiologic factors (Table 3). In the univariable and multivariable analysis, the number of radiologic viable tumour ≤ 1, maximum diameter of viable tumour ≤ 1 cm, and AFP ≤ 20 ng/mL were independently predictive of PDS. The MELD score, history of liver resection, and the number and modality of LRT were not associated with PDS.

**Table 3:** Pretransplant factors associated with pathologic downstaging.

Variables	Univariate		Multivariate^a^	
	OR (95% CI)	*P*	OR (95% CI)	*P*
Radiologic findings				
Number of viable tumors ≤ 1	3.95 (1.97–7.94)	<0.001	2.77 (1.29–5.94)	0.009
Maximum diameter of viable tumor ≤ 1 cm	3.74 (2.08–9.72)	<0.001	3.31 (1.56–7.03)	0.002
AFP ≤ 20 ng/mL	3.41 (1.66–7.02)	0.001	2.52 (1.32–4.83)	0.005
MELD ≤ 20	0.54 (0.23–1.28)	0.163		
History of liver resection for HCC	0.68 (0.35–1.34)	0.268		
Number of LRT	0.96 (0.84–1.09)	0.540		
**Modality of LRT**				
TACE, only	Reference			
RFA, only	2.07 (0.87–4.99)	0.102		
TACE plus RFA	1.61 (0.79–3.25)	0.188		
Others	1.64 (0.26–10.13)	0.597		

^a^Multivariate analysis was performed by logistic regression.

AFP alpha fetoprotein; HCC hepatocellular carcinoma; MELD, model for end-stage liver disease; LRT, locoregional treatment; RFA, radiofrequency ablation; TACE, trans arterial chemoembolization; TN, total necrosis.

### Posttransplant outcomes

**Figure 2:**
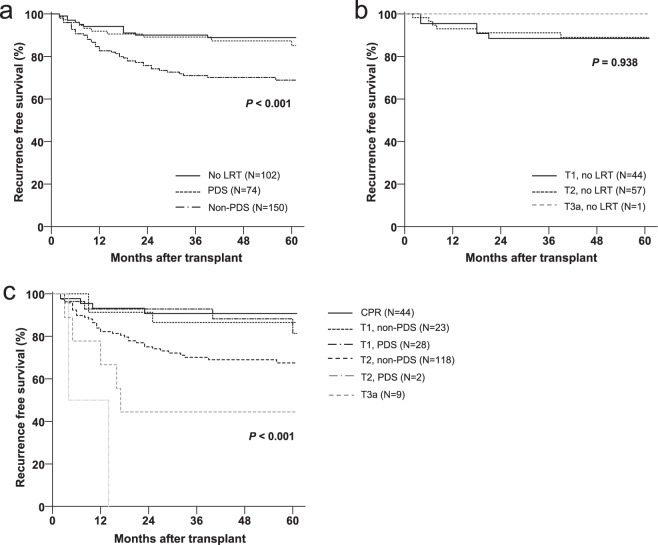
Comparison of HCC recurrence free survival; (**a**) by LRT and PDS status; (**b**) by T stages among the patients who did not receive LRT; (**c**) by T stage and PDS status among the patients who received LRT. HCC, hepatocellular carcinoma; LRT, locoregional treatment; PDS, pathologic downstaging.

Five-year recurrence free survival (RFS) of entire cohort was 78.9%. PDS group showed similar RFS with no LRT group but non-PDS group showed worse prognosis (88.8% vs. 85.1% vs. 68.9% for no LRT, PDS, and non-PDS respectively, *P* < 0.001) (Fig. 2a). We compared outcomes according to each T stage both in the patients who received LRT and who did not. In no LRT group, post-transplant outcomes were similar between T1 and T2 cancer (88.5% vs. 89.0% for T1 and T2 respectively, *P* = 0.938) (Fig. 2b). Patient with T3 cancer was only 1 and did not experience recurrence or death. In contrast, RFS and HCC recurrence were well stratified according to T stage adjusted by the exclusion of TN in the patients received LRT (90.7% vs. 83.1% vs. 67.3% vs. 50.0% for CPR, T1, T2 and T3a respectively, *P* < 0.001).

Figure 2c shows the comparison of outcomes according to the adjusted T stage and PDS status. As expected, CPR showed the best RFS (90.7%). PDS T1 (82.4%) showed higher RFS than non-PDS T2 (67.5%), and similar RFS with non-PDS T1(86.5%). T3a cancers showed much worse prognosis (44.4%) than the formers. However, patients with PDS T2 HCC were only 2 and both of them experienced HCC recurrence.

**Figure 3:**
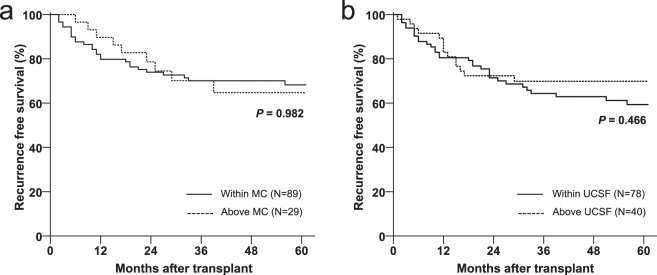
Comparison of HCC recurrence free survival (**a**) by radiologic Milan criteria at the time of transplantation and (**b**) by UCSF criteria on explanted liver pathology among the patients with non-PDS T2 cancers HCC, hepatocellular carcinoma; PDS, pathologic downstaging; MC, Milan criteria; UCSF, University of California San Francisco.

For subgroup survival analysis, patients with non-PDS T2 HCC were divided into two groups according to Milan criteria by radiology or University of California San Francisco criteria (UCSF) criteria by explant pathology, respectively. However, RFS was similar in non-PDS T2 cancers regardless of Milan criteria (*P* = 0.982) or UCSF criteria (*P* = 0.466) (Fig. 3).

## Discussion

LRT has been widely performed as a bridging therapy before LT in HCC patients because the majority of LT candidates present with more advanced stages than the Milan criteria^11^. However, post-LT outcomes after LRT were not concordant among studies because the study populations and criteria for LRT were heterogenous^12^. Although two prospective studies showed that LRT for properly selected patients resulted in comparable outcome to conventional criteria^5,7^, the exact prediction of HCC recurrence based on liver pathology is still necessary. In addition to the global consensus in which there is no consideration for the effect of LRT^6^, the current study tried to demonstrate that pathologic downstaging by the exclusion of TN was a favourable prognostic factor after liver transplantation for HCC.

The most remarkable finding of our study was that the patients achieving PDS showed better RFS than those in the non-PDS group, and showed similar RFS with those in the no LRT group, about 90% of whom were within the Milan criteria. Especially, the outcomes of PDS T1 cancer were significantly better than those of non-PDS T2 and similar as those with non-PDS T1 and no LRT T1. These results mean that PDS T1 could be considered as original T1 in terms of tumour recurrence. Non-PDS T2 comprises patients who failed to demonstrate reduced tumour stage owing to multiple viable tumours or solitary tumour with MVI. These non-PDS T2 showed worse outcomes, regardless of the Milan criteria or UCSF criteria, which were conventional preoperative or postoperative predictors for HCC prognosis.

Several studies emphasized on the pathologic response of LRT before LT. Previously, two studies reported that the degree of tumour necrosis from pre-transplant LRT was associated with HCC recurrence and survival^13,14^. However, Agopian *et al*.^15^ reported that CPR was a strong predictor of tumour-free survival. Allard *et al*.^16^ also showed that >90% total proportion of necrosis was an independent factor for better prognosis. Furthermore, more recent studies alluded that patients who did not achieve CPR had worse prognosis than those who did not receive LRT before LT^17,18^. The evidence of the unfavourable outcomes of partial response was that cancer cells could be disseminated by LRT, unless they were completed destroyed. However, from this study, we demonstrated that patients with PDS T1 also showed good prognosis, although they were “partial response group”.

Otto *et al*.^19^ reported that tumour response to TACE was a more reliable factor for selecting LT candidate than size and number themselves. This concept based on tumour behaviour and biology is advocated by numerous studies^15–18,20–23^. Kim *et al*.^24^ demonstrated that patients with considerable response to LRT had less frequent vascular invasion and poor differentiation. Although radiologic tumour information before LRT was not available in our dataset, tumour behaviour could be inferred from the fact that the number of TN was higher in the PDS group than that in the non-PDS group, in spite of similar LRT number and modality. Lower AFP at the time of LT was another evidence of good tumour behaviour in the PDS group.

Because of organ shortage, liver transplantation from living donor is gaining importance not only in Asia but also in the western countries^25^; however, it could not always be possible at the presentation of HCC patients. Eventually, majority of patients receive LRT as the first treatment option. In this situation, our study suggests that PDS could be targeted by pretransplant LRT for excellent posttransplant outcome. Although it has a limitation that MVI cannot be examined preoperatively, our data suggested that tumour number and size on the imaging study along with AFP could predict PDS.

Interpretation of the impact of PDS was limited in CPR and PDS T1 cancers in this study because of the small number of PDS T2 (n = 2). The possible explanation is that original T3 cancer would present aggressive biology and would be difficult to induce complete necrosis of individual mass. Further studies using larger volume data are needed to assess the prognosis of PDS T2.

In conclusion, the current study demonstrated that the outcome of PDS group was better than that of the non-PDS group and similar with that of no the LRT group after liver transplantation. Furthermore, subgroup analysis confirmed that PDS T1 had favourable outcome like the original T1 HCC. These results could help clinicians determine the treatment modality before LT and predict HCC recurrence more accurately during the post-transplant period.

## Methods

### Patients

We conducted a retrospective, single centre LT data base analysis on 792 patients who received LT from September 2005 to December 2016. Among 727 patients aged over 18 years, 391 had HCCs in their own livers. Indications of LT for HCC in our institution were; (1) Unresectable HCC within the MC, (2) HCC initially beyond the MC but downstaged to meet the MC after LRT, (3) HCC with severe underlying LC regardless of resectability and prior LRT. Patients whose serum AFP was rising were not considered as a candidate for LT, although they meet the MC on radiologic studies.

We included only HCC patients diagnosed based on the explant liver pathology, including incidental HCC. Patients with mixed HCC with cholangiocellular carcinoma (n = 19) and HCC patients treated with chemo-radiation therapy before LT (n = 26), and those who died within 1 month after transplant (n = 20) were excluded. Finally, 326 recipients were analysed.

### Diagnosis of hepatocellular carcinoma and locoregional treatment before liver transplantation

Diagnosis of HCC was based on imaging studies as recommended in the recent guidelines^26^. We performed LRT as a bridging therapy for most of HCCs beyond the Milan criteria. Modalities of LRT were categorized as only TACE or RFA, TACE plus RFA, and miscellaneous treatments, including chemical ablation and transarterial radioembolization. The Milan criteria were re-assessed at the time of LT after LRT.

### Pathologic findings and hepatocellular carcinoma staging

From explant liver pathology, tumours that contained no viable cancer cells were defined as TN. Remainder in which even single cancer cell was detected by H&E stain were recorded as viable tumour. The number of viable tumours and TN as well as the diameter, MVI, and tumour differentiation of each were recorded. We staged each HCC patient using the TNM system 7^th^ edition^27^. For patients who received pre-transplant LRT, T stage including the totally necrotic mass was considered as the original stage before LRT, whereas that excluding the totally necrotic mass was considered as the final stage after LRT. Thereafter, the study population was divided into no LRT, PDS (reduced T stage by the exclusion of TN), and non-PDS groups (unchanged T stage despite the exclusion of TN).

### Patient follow up

AFP was checked every two or three months, and liver dynamic computed tomography (CT) scan was done in cases with increased AFP. Although AFP was normal, abdominal and chest CT scan were done at least every year for early detection of HCC recurrence. The primary endpoints were RFS after LT.

### Statistical analysis

Data are presented as number (percentage) for categorical variables and median (IQR) for continuous variables. The chi square test, Mann-Whitney U test, and Kruskal-Wallis test were used for comparison between groups as appropriate. RFS was estimated using the Kaplan-Meier survival curve and compared using the log rank test between groups. To define the pretransplant predictive factors for pathologic downstaging, univariable and multivariable logistic regressions were performed. All analyses were conducted using the SPSS software (SPSS v23.0; IBM, Armonk, NY, USA)), and *P* values < 0.05 were considered statistically significant.

### Ethic statement

The study was performed according to the principles of the Declaration of Helsinki and the Declaration of Istanbul. It was approved by the independent Institutional Review Board of the Yonsei University College of Medicine (IRB No.: 4-2018-0987), which waived the need to obtain informed consent due to the retrospective nature of this study.

## Data Availability

The datasets for this study are available from the corresponding author on the reasonable request.
